# A Novel Diagnostic Biomarker, PZP, for Detecting Colorectal Cancer in Type 2 Diabetes Mellitus Patients Identified by Serum-Based Mass Spectrometry

**DOI:** 10.3389/fmolb.2021.736272

**Published:** 2021-11-30

**Authors:** Jiayue Yang, Weigang Fang, Wenjun Wu, Zhen Tian, Rong Gao, Lu Yu, Dayang Chen, Xiaohua Weng, Shengwei Zhu, Cheng Yang

**Affiliations:** ^1^ Department of Endocrinology, The Affiliated Wuxi People’s Hospital of Nanjing Medical University, Wuxi, China; ^2^ Department of Gastroenterology, The Affiliated Wuxi People’s Hospital of Nanjing Medical University, Wuxi, China; ^3^ Department of Clinical Laboratory, The Affiliated Wuxi People’s Hospital of Nanjing Medical University, Wuxi, China

**Keywords:** biomarker, colorectal cancer, type 2 diabetes mellitus, mass spectrum, PZP

## Abstract

**Background:** Growing evidence has confirmed that populations with type 2 diabetes mellitus (T2DM) have an increasing risk of developing colorectal cancer (CRC). Thus, convenient and effective screening strategies for CRC should be developed for the T2DM population to increase the detection rate of CRC.

**Methods:** Twenty serum samples extracted from five healthy participants, five T2DM patients, five CRC patients and five T2DM patients with CRC (T2DM + CRC) were submitted to data-independent acquisition mass spectrometry (DIA-MS) analysis to discover unique differentially altered proteins (DAPs) for CRC in patients with T2DM. Then, the diagnostic value of pregnancy zone protein (PZP) was validated by ELISA analysis in the validated cohort.

**Results:** Based on DIA-MS analysis, we found eight unique proteins specific to T2DM patients with CRC. Among these proteins, four proteins showed different expression between the T2DM + CRC and T2DM groups, and PZP exhibited the largest difference. Next, the diagnostic value of serum PZP was validated by ELISA analysis with an AUC of 0.713. Moreover, the combination of PZP, CA199 and CEA exhibited encouraging diagnostic value, and the AUC reached 0.916.

**Conclusion:** Overall, our current research implied that PZP could be regarded as a newfound serum biomarker for CRC medical diagnosis in T2DM patients.

## Introduction

Diabetes mellitus is a widespread chronic disease, especially type 2 diabetes mellitus (T2DM), whose incidence is increasing gradually. According to the latest statistics published by the International Diabetes Federation (ninth edition), the global diabetes prevalence in 2019 was expected to be 9.3%, which will increase to 10.2% by 2030 and 10.9% by 2045 (Saeedi et al., 2019). In China, the estimated prevalence of diabetes was expected to be 11.6% in the adult population, and the prevalence of prediabetes was 50.1% (Xu et al., 2013). In addition, T2DM accounts for 90–95% of total diabetes cases (DeFronzo et al., 2015). Increasing studies have demonstrated that T2DM is associated not only with micro- and/or macrovascular complications (DeFronzo et al., 2015) but also with carcinogenesis and progression of various types of malignancies, such as lung cancer, gynecological cancers and gastrointestinal cancers (Shlomai et al., 2016; Abudawood, 2019; Pearson-Stuttard et al., 2021).

Colorectal cancer (CRC) is one of the most widespread malignant tumors in the digestive system; the third highest incidence rate of all cancers and ranks as one of the cancers with the highest mortality rates (Siegel et al., 20192019). Because the prognosis of CRC primarily depends on the tumor stage, early detection plays a decisive role in the treatment and prognosis of CRC patients (Messersmith, 2019). However, a large number of patients are still diagnosed at progressive stages, and the 5-years survival rate is only approximately 30%, even after standardized systemic treatment (Siegel et al., 2020). Increasing evidence has confirmed that T2DM patients face an enhanced threat of suffering from CRC compared to a population without the disease (Larsson et al., 2005; Elwing et al., 2006; Yu et al., 2016). Therefore, the T2DM population should be regarded as a high-risk population prone to CRC, and convenient and effective screening strategies for CRC should be developed for the T2DM population to increase the detection rate of CRC and improve the prognosis of these patients.

In this study, data-independent acquisition mass spectrometry (DIA-MS) was used to characterize serum protein profiles of healthy participants, T2DM patients, CRC patients and T2DM patients complicated with CRC (T2DM + CRC). In addition, the unique differentially altered proteins (DAPs) in the serum of T2DM + CRC patients were determined, and one of these proteins, PZP was further verified by enzyme-linked immunosorbent assay (ELISA) analysis in the validated cohort. In summary, pregnancy zone protein (PZP) was identified as a newfound serum indicator for monitoring CRC in patients with T2DM, which will boost the noninvasive diagnosis rate for CRC in clinical practice.

## Materials and Methods

### Patients and Sample Description

**FIGURE 1:**
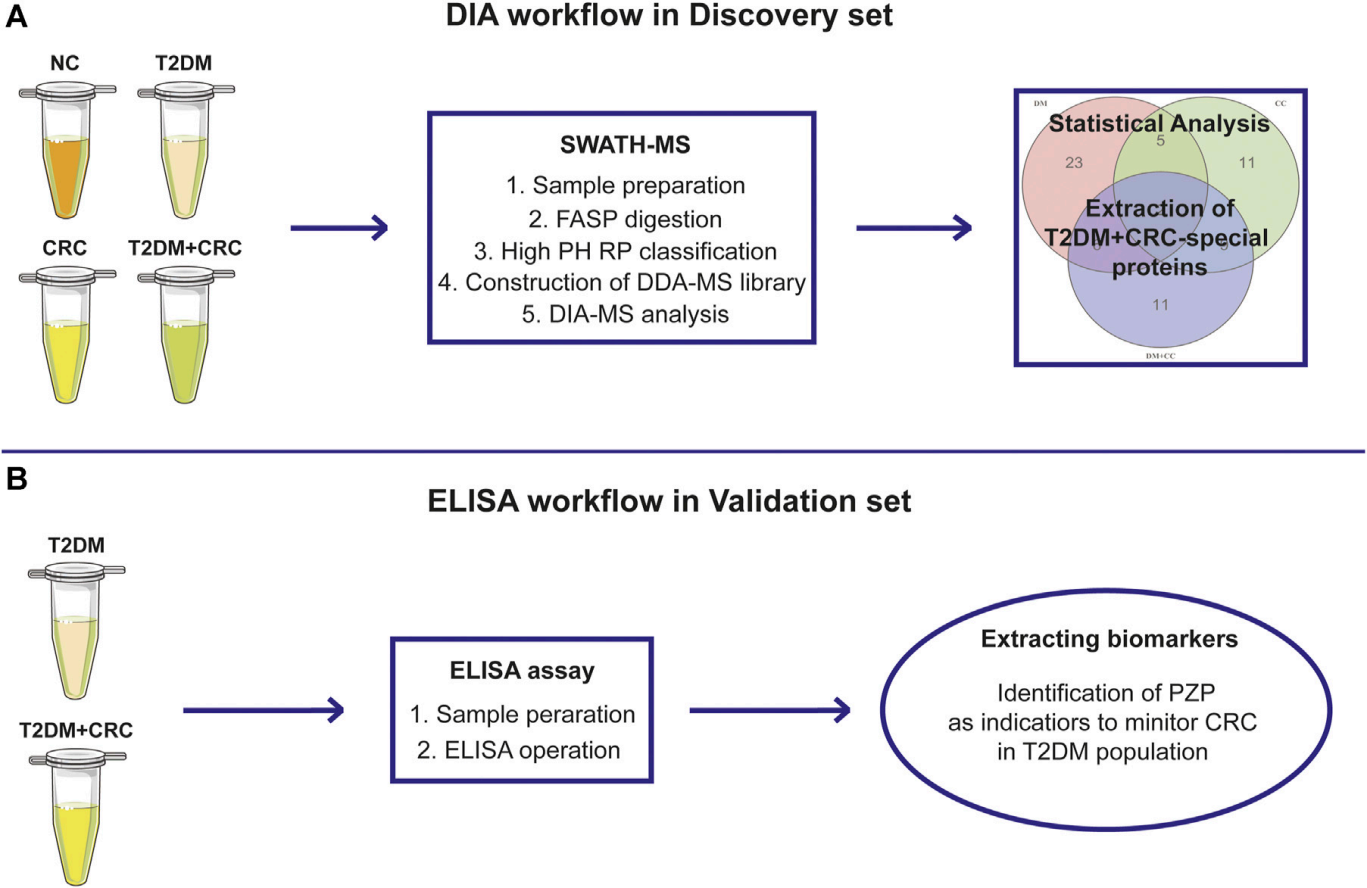
The overall design of the current study. Schematic illustration of the discovery procedure for the screening of potential diagnostic biomarkers for CRC in T2DM patients based on the DIA-MS method **(A)** and the validated procedure based on ELISA analysis **(B)**.

The following two patient cohorts were recruited to identify and verify serum indicators for screening CRC in T2DM patients (Figure 1): In the discovery cohort, a total of 20 serum samples from five healthy participants, five T2DM patients, five CRC patients and five T2DM + CRC patients, were collected for DIA-MS analysis. The baseline information in the discovery cohort was exhibited in Supplementary Table S1. In the validation cohort, 40 serum specimens from T2DM patients and 32 serum samples from T2DM + CRC patients were submitted to ELISA analysis. The baseline information of patients in the validation cohort was exhibited in Table 1. Before collecting serum samples, patients or healthy participants did not receive any treatment other than hypoglycemic therapy. The serum specimens were preserved at -80 °C until use. The general information of the patients and the results of tumor biomarkers (AFP, CEA, CA125 and CA199) were obtained by retrospective review of medical records. The Ethical Committee at Wuxi People’s Hospital Affiliated to Nanjing Medical University gave the ethical license to the study.

**TABLE 1:** Comparison of general information between two groups.

General information	T2DM	T2DM + CRC	*p* Value
Sex (male/female)	23/17	15/17	0.370
Age (years)	61.05 ± 9.78	68.69 ± 8.87	0.001
AFP (ng/ml)	2.70 ± 1.17	2.30 ± 0.72	0.311
CEA (ng/ml)	2.11 ± 1.09	19.31 ± 63.44	<0.001
CA125 (U/ml)	9.50 ± 4.86	14.69 ± 24.12	0.377
CA199 (U/ml)	14.41 ± 10.44	149.10 ± 451.3	0.016
FPG (mmol/L)	7.95 ± 1.91	6.53 ± 2.09	0.004

### DIA-MS Analysis

DIA-MS analysis was performed on each serum sample from the discovery set to obtain differential serum proteins. The main steps were as follows: protein extraction and FASP enzymatic hydrolysis, high PH RP classification, DDA-MS library construction and DIA analysis, these techniques were described in our previous publication (Yang et al., 2021). DAPs were extracted using R language 4.0.0 with a criterion of fold change (FC) ≥ 1.50 or ≤0.67 and a unpaired *t*-test *p* value ≤0.05.

### ELISA Analysis

The serum levels of PZP were quantified using a commercial ELISA kit (Cat no. DY8280-05; R&D Systems) based on the manufacturer’s protocol. An 8-point standard curve was fitted to the OD values of each standard sample after subtraction of the blank sample. The concentration of PZP in serum was reached using the regression equation and multiplied by the dilution ratio (with a dilution of 1:2). All specimens were examined in duplicate, and the average levels were used in all analyses in this research.

### Statistical Analysis

Most statistical analyses were performed using SPSS (version 26.0) and GraphPad Prism (version 8.0). Figure exhibition was performed using R language 4.0.0 and GraphPad Prism (version 8.0). The difference between the two groups was assessed by Student’s *t*-test or Mann-Whitney test. Receiver operating characteristic (ROC) analysis was plotted to evaluate the specificity and sensitivity of the candidate indicator, and the area under the ROC curve (AUC) was generated for diagnostic biomarkers. Binary logistic regression analysis was devoted to evaluating the combined diagnostic performance. For all analyses, a *p* value ≤0.05 was considered to be statistically significant.

## Results

### Clinical Features of T2DM and T2DM + CRC Patients

The general clinicopathological characteristics in the two groups in the validation cohort consisting of 40 T2DM patients and 32 T2DM + CRC patients were compared. Of the 40 patients in the T2DM group, 23 were males and 17 were females, with an average age of 61.05 ± 9.78 years and an average fasting plasma glucose (FPG) of 7.95 ± 1.91 mmol/L. In the T2DM + CRC group, there were 15 males and 17 females with an average age of 68.69 ± 8.87 years and an average FPG of 6.53 ± 2.09 mmol/L. No statistically significant difference was found between the sex of the two groups (*p* = 0.370), but the differences in age (*p* = 0.001) and FPG (*p* = 0.004) were statistically significant (Table 1). Next, we evaluated the differences in the levels of the widely applied tumor biomarkers between these two groups. The results showed that there were no remarkable differences in serum AFP (*p* = 0.311) and CA125 (*p* = 0.377) levels between the T2DM + CRC group and the T2DM group, but CEA (*p* < 0.001) and CA199 (*p* = 0.016) levels were notably enhanced in the T2DM + CRC group (Table 1).

### Discovery of Differentially Expressed Proteins by DIA-MS Analysis

**FIGURE 2:**
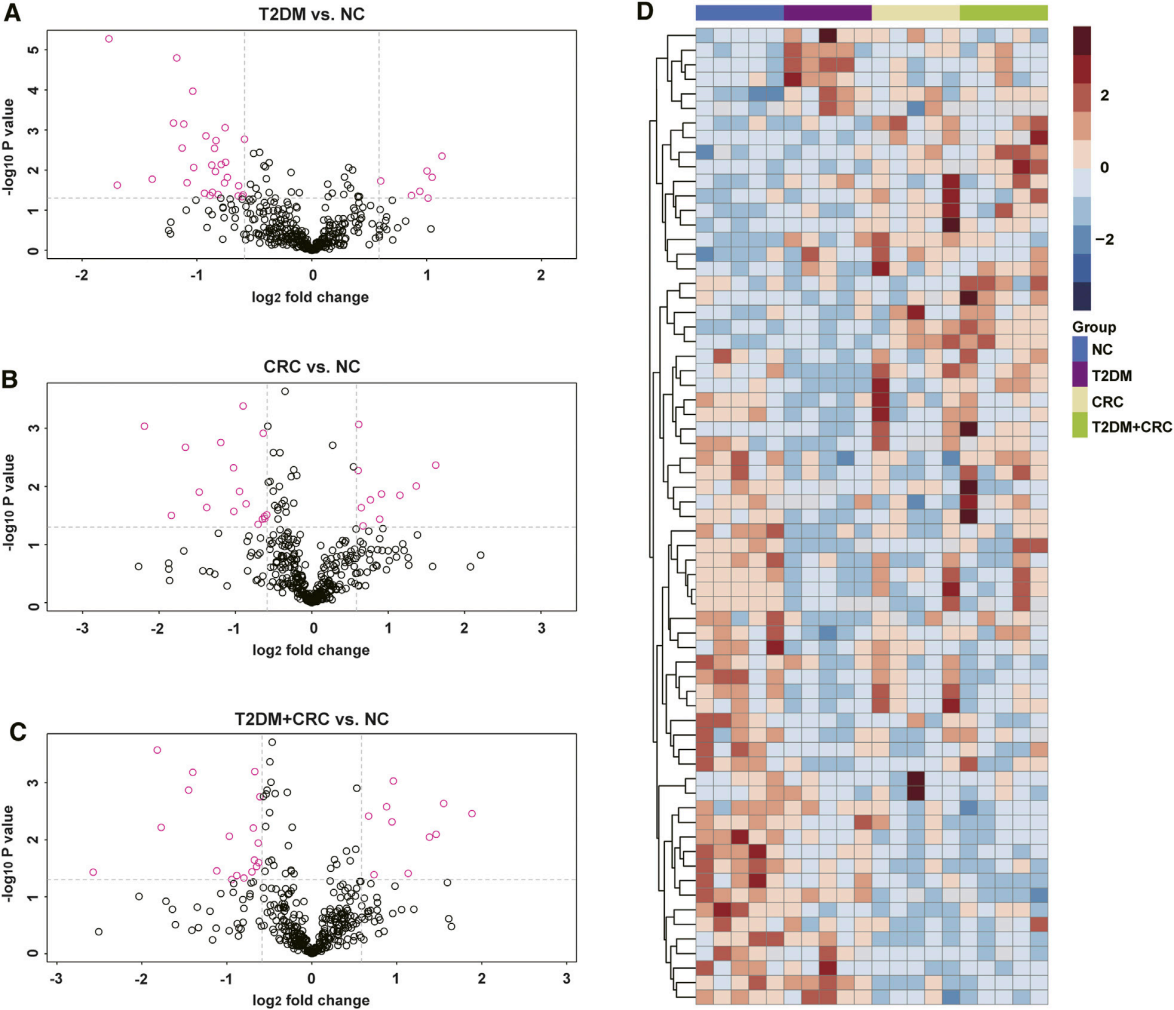
Serum protein profiles in patient cohorts in the current study. **(A–C)** Volcano plot exhibiting the DAPs in the T2DM, CRC and T2DM + CRC patients in comparison with healthy participants. DAPs were extracted using R language 4.0.0 with a criterion of fold change (FC) ≥ 1.50 or ≤0.67 and unpaired *t*-test *p* value ≤0.05. **(D)** Hierarchical cluster analysis of the 67 DAPs in the groups of healthy participants, T2DM, CRC, and T2DM + CRC. There were five samples in each group. Red represents for high expression and blue represents for low expression.

**FIGURE 3:**
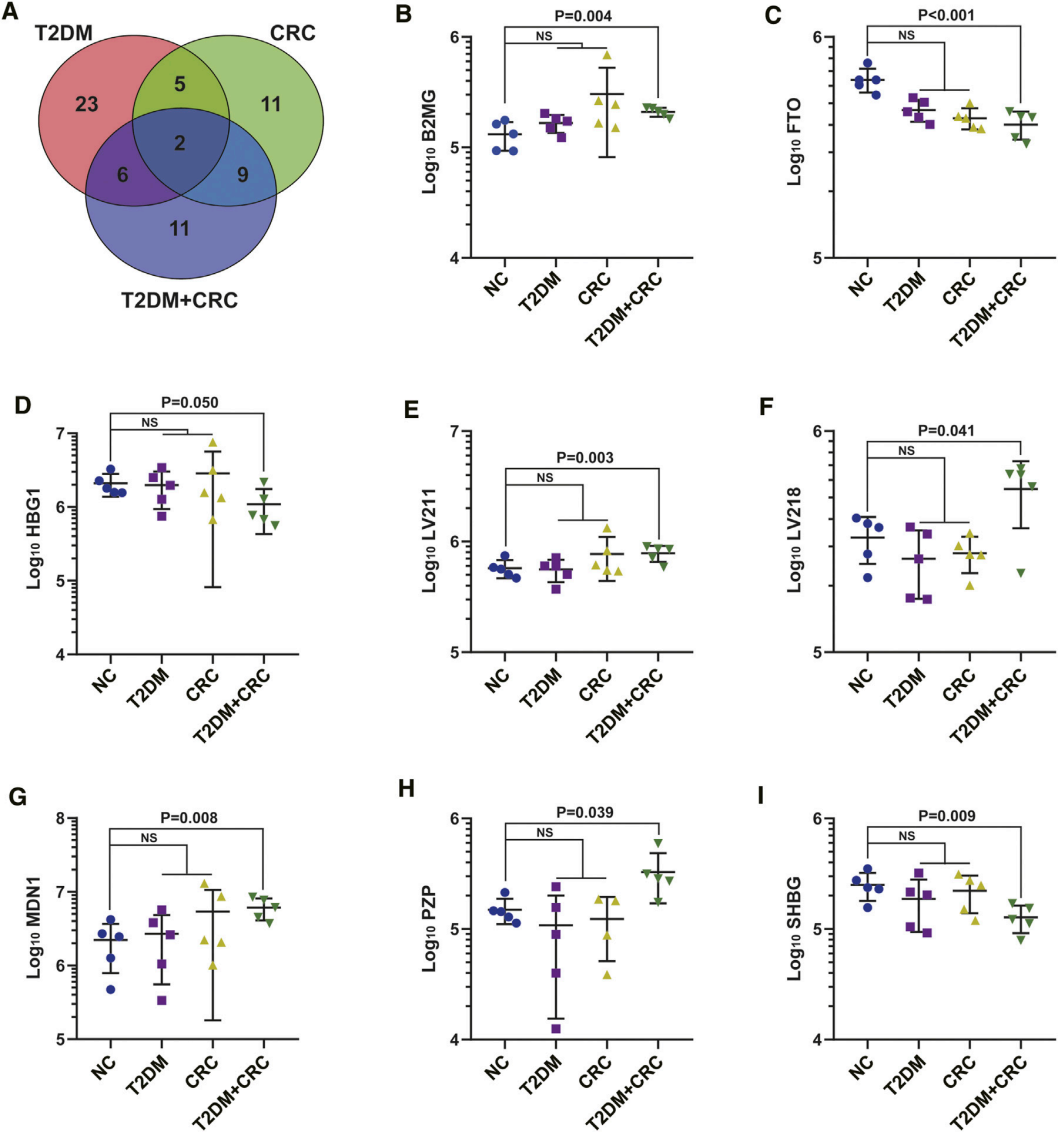
Extraction of specific serum proteins in T2DM + CRC patients. **(A)** Venn diagram of the 67 DAPs in the serum specimens from the four comparisons (T2DM vs healthy participants, CRC vs healthy participants and T2DM + CRC vs healthy participants). **(B–I)** Statistical graphs of eight specific proteins levels. Unpaired *t*-test was used to compare the potential difference.

Based on DIA-MS analysis, the overall protein changes in serum specimens from 20 patients (5 healthy participants, five T2DM patients, five CRC patients and five T2DM + CRC patients) were analyzed. The levels of 67 serum proteins were deemed remarkably different between these disease groups and the healthy participant group (Figures 2A–C). To redistribute the specimens based on similarities in the patterns of serum protein levels, we conducted a hierarchical cluster analysis of the 67 DAPs according to previous research (Huang et al., 2010; Yang et al., 2021). Cluster analysis revealed a clear separation of the four groups (Figure 2D). Next, three protein subgroups from the afore mentioned analysis (T2DM vs healthy participant, CRC vs healthy participant and T2DM + CRC vs healthy participant) were differentiated to identify a subgroup of proteins that were differentially expressed only in the T2DM + CRC group. In total, eight distinct proteins were regarded as specific candidates in patients with T2DM + CRC (Figures 3A–I). Furthermore, the levels of four candidates were remarkably different between the T2DM + CRC and T2DM groups, namely, B2MG (FC = 1.257, *p* = 0.031), LV218 (FC = 2.067, *p* = 0.015), MDN1 (FC = 2.274, *p* = 0.032) and PZP (FC = 3.032, *p* = 0.027), and PZP exhibited the largest discrimination level (Table 2).

**TABLE 2:** Unpaired *t*-test of the quantitative proteomic results for protein expression in the T2DM + CRC group in comparison with the control and T2DM groups.

Proteins	T2DM + CRC vs NC		T2DM + CRC vs T2DM	
	Fold change	*p* Value	Fold change	*p* Value
B2MG	1.589	0.004	1.257	0.031
FTO	−1.592	0.000	−1.162	0.101
HBG1	−1.924	0.050	−1.822	0.144
KIF5A/KIF5C/KINH^a^	−1.543	0.025	−1.423	0.004
KRT81/KRT83/KRT85/KRT86^a^	2.611	0.009	1.730	0.017
LV211	1.840	0.003	1.563	0.129
LV218	1.660	0.041	2.067	0.015
MDN1	2.757	0.008	2.274	0.032
PZP	2.194	0.039	3.032	0.027
SHBG	−1.961	0.009	−1.469	0.217
ZGRF1/YD002^a^	−1.527	0.002	−1.364	0.084

NC, healthy participant.

a These proteins were excluded from subsequent analysis due to their nonspecificity.

### Validation of the Diagnostic Value of PZP and Combined Diagnostic Analysis

**FIGURE 4:**
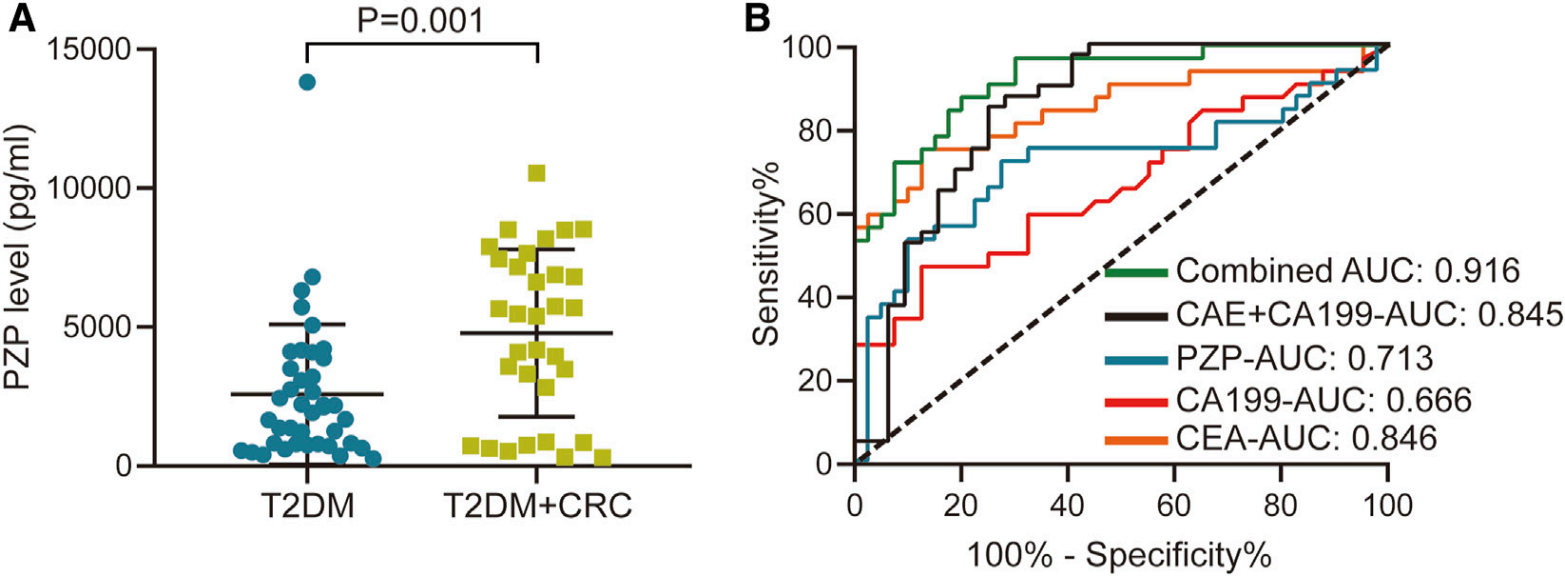
Diagnostic value of PZP and combined diagnostic analysis. **(A)** Serum levels of PZP in the T2DM and T2DM + CRC groups. Unpaired *t*-test was used to compare the potential difference. **(B)** ROC analysis of the diagnostic values of PZP, CA199, CEA and the combined score.

We subsequently validated the changes in PZP protein abundance using a commercial ELISA kit. The concentration-dependent standard curve is shown in Supplementary Figure S1. In addition, the level of PZP between the T2DM + CRC and T2DM groups was notably different in the validated cohort (Figure 4A), and the ROC analysis suggested an AUC of 0.713 (Figure 4B). To exclude the influence of confounding factors on PZP level, including age and sex, we compared the PZP level in male and female participants and assessed its correlation with age. There were no notable differences in PZP levels between males and females (*p* = 0.192), and no notable correlation was observed between PZP levels and patient age (*p* = 0.179) (Supplementary Figure S2). Considering the notable diagnostic value of CA199 and CEA in identifying CRC in T2DM populations, we next conducted a combined diagnostic performance test. Encouragingly, the combination of PZP, CA199 and CEA exhibited excellent diagnostic value compared with CA199, CEA and combined CA199&CEA, and the AUC reached 0.916 (Figure 4B).

## Discussion

Previous studies have shown that T2DM patients have a superior risk of suffering from CRC in comparison with the general population (Yuhara et al., 2011; Sinagra et al., 2017). Hsieh et al. (Hsieh et al., 2012) previously examined the impact of T2DM on cancer risk using a logistic regression model. They found that T2DM patients have a notably higher risk of CRC. Chiu et al. (Chiu et al., 2013) applied the Cox proportional hazard regression model to assess the impacts of T2DM on the morbidities of gastrointestinal malignancies. They pointed out that T2DM was correlated remarkably with an enhanced threat of developing CRC. In addition, studies have shown that the application of the hypoglycemic drug metformin could significantly decrease the risk of CRC (Chen et al., 2020). Apart from this, metformin having the ability to mediate alterations to multiple critical events, including CRC cell proliferation, stemness maintenance, epithelial-mesenchymal transition (EMT), and transformational protumor cellular metabolic conditions (Jaromy and Miller, 2021). Moreover, CRC patients with T2DM receiving medical treatment with metformin experienced fewer distant metastases, associated with slower progression of CRC (Powell et al., 2020). A retrospective study pointed out that patients in the T2DM + CRC group were older than those in the T2DM group in a randomly selected cohort, which was consistent with our research (Feng et al., 2020). Therefore, it is very important to carry out CRC screening in elderly T2DM patients.

With the development of medical technology, there are increasing numbers of strategies available for CRC screening, consisting of both invasive strategies (colonoscopy, sigmoidoscopy and capsule colonoscopy) and noninvasive strategies (stool DNA testing and blood tumor biomarker testing) (Nee et al., 2020). Accumulating evidence based on observational studies has suggested that colonoscopy screening has a remarkably high success rate resulting in decreased (Manser et al., 2012; Nishihara et al., 2013; Brenner et al., 2014). The risk of T2DM patients developing CRC is significantly increased, and regular screening should be carried as they are a high-risk group. The latest research pointed out that through colonoscopy, the detection rate of colonic adenoma in T2DM patients was notably higher than that of the general population (Ottaviano et al., 2020). Although colonoscopy and other invasive screening modalities are generally regarded as safe, the threats of bowel preparation, the procedure, and sedation medications are all enhanced in older patients (Nee et al., 2020). In addition, colonoscopies are very difficult to carry out, especially in primary hospitals, due to the high technical requirements of staff, low patient acceptance, and high medical costs. Therefore, it is very important to find noninvasive diagnostic methods, especially serum biomarkers, to accurately identify CRC in T2DM patients.

The discovery and validation of tumor biomarkers involve a variety of modalities and platforms. Among them, MS-based strategies are widely utilized (Whetton et al., 2020; Ponzini et al., 2021). DIA-MS is a fairly simplified and convenient method to conduct high-throughput, quantitative and highly renewable proteomic analyses of clinical specimens (Guo et al., 2015). Based on the DIA-MS method, an increasing number of novel tumor indicators have been progressively discovered (Ortea et al., 2016; Ku et al., 2020). In the current research, to identify effective serum biomarker for CRC in T2DM population, we characterized the serum protein profiles of healthy participants, T2DM patients, CRC patients and T2DM + CRC patients. We identified a subgroup of proteins that were differentially expressed specifically in the T2DM + CRC group. A total of eight precise proteins were found to be specific to T2DM + CRC patients. In addition, the levels of four candidate proteins were prominently different between the T2DM + CRC and T2DM groups, and PZP exhibited the largest difference. To further validate the diagnostic value of PZP in screening CRC in T2DM populations, the validated validation cohort consisting of 40 T2DM patients and 32 T2DM + CRC patients was applied, and the results confirmed that promising diagnostic value.

PZP has been regarded as a pregnancy-associated protein that is remarkably overexpressed in the decidua of recurrent and spontaneous miscarriage (Lin and Halbert, 1976; Lob et al., 2021). Previous research suggested that serum PZP levels are notably elevated in patients with ovarian cancer in comparison with those with benign ovarian tumors and in those with cervical or endometrial cancer in comparison with those with benign uterine myomas (Teng et al., 1994). PZP has been identified as an unprecedented noninvasive indicator for screening lung adenocarcinoma in T2DM in our previous research (Yang et al., 2021). Furthermore, several tumor biomarkers already in clinical use also exhibited valuable discriminatory activity in screening CRC, the combination of PZP, CA199 and CEA exhibited encouraging diagnostic value, and the AUC reached 0.916.

In addition, the current research also provided a clue for the discovery of general CRC biomarkers. Namely, these nine proteins shared between the T2DM-CRC and CRC groups may be effective biomarkers for CRC regardless of diagnosing with T2DM or not. As shown in Supplementary Figure S4, we could found that FUBP1/2, PON3 and FHR1 were increased in general CRC (CRC and T2DM + CRC) patients compared with non-CRC patients (NC + T2DM), while KV104 and FRPD2 were decreased (Supplementary Figure S4). Recent research reported that FUBP1 promoted CRC stemness and metastasis by activating Wnt/β-catenin signaling (Yin et al., 2021). Whether increased FUBP1 was derived from tumor tissues and whether FUBP1 could be potential biomarker for CRC diagnosis needed to be further studied.

Whatever, there were some limitations in the current study. The major limitation is the small sample size. The patients diagnosed with T2DM + CRC are not common in our hospital but could not be ignored on a larger scale. Thus, multi-centric observational studies with a larger sample size should be conducted before PZP could be applied in clinical practice. Besides, in our previous research, PZP was identified as a serum indicator for screening lung adenocarcinoma in T2DM. We also compared the serum level of PZP in T2DM, T2MD + CRC and T2DM with lung adenocarcinoma (T2MD + LAC) patients, and the results showed that PZP was increased in T2MD + CRC and T2MD + LAC patients, but no difference was observed in these two groups (Supplementary Figure S3). Thus, whether PZP is a pan-cancer or pan-adenocarcinoma biomarker in T2DM needs to be further explored.

## Conclusion

In summary, we successfully identified PZP as a potential diagnostic classifier for CRC screening in T2DM patients through DIA-MS combined with ELISA. In addition, the combined application of PZP with conventional tumor biomarkers CA199 and CEA has stronger diagnostic efficiency. However, the above results still need to be verified by multiple centers and larger samples.

## Data Availability

The original contributions presented in the study are included in the article/Supplementary Material, further inquiries can be directed to the corresponding authors.
