# Supplementary material for: A Novel Diagnostic Biomarker, PZP, for Detecting Colorectal Cancer in Type 2 Diabetes Mellitus Patients Identified by Serum-Based Mass Spectrometry
Source: Front Mol Biosci. 2021 Nov 30;8:736272. doi: 10.3389/fmolb.2021.736272 (PMC8670180; doi:10.3389/fmolb.2021.736272)
Supplement: Supplementary file 1 [file Presentation1.ZIP › Fig S1.docx]

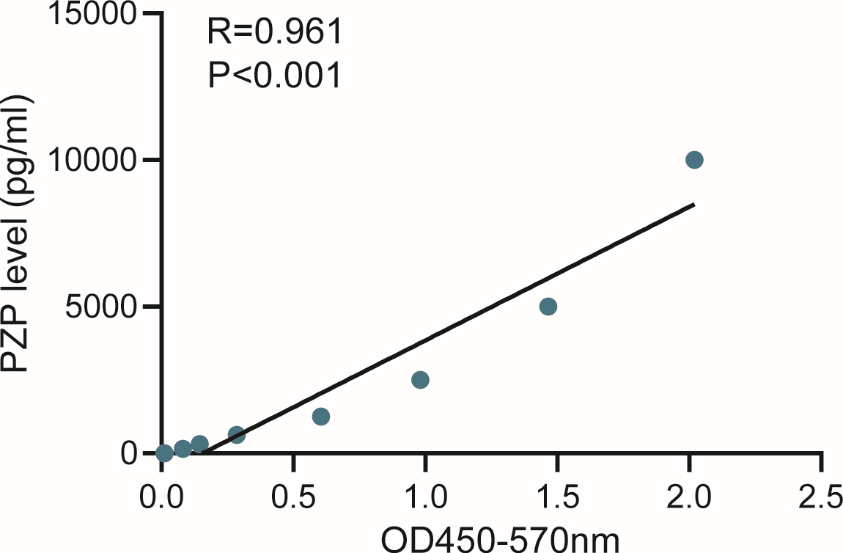


Figure S1. Standard curve of ELISA assay for Pearson analysis was used to assess the correlation between OD450-570 and PZP level.
