# Supplementary material for: A Novel Diagnostic Biomarker, PZP, for Detecting Colorectal Cancer in Type 2 Diabetes Mellitus Patients Identified by Serum-Based Mass Spectrometry
Source: Front Mol Biosci. 2021 Nov 30;8:736272. doi: 10.3389/fmolb.2021.736272 (PMC8670180; doi:10.3389/fmolb.2021.736272)
Supplement: Supplementary file 1 [file Presentation1.ZIP › Fig S2.docx]

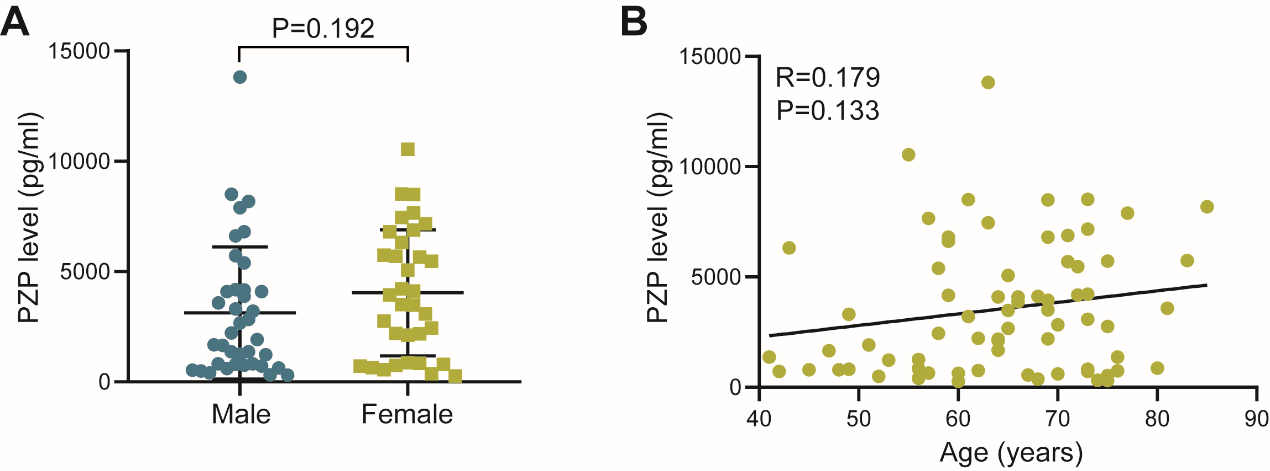


Figure S2. (A) PZP level in male and female participants and (B) its correlation with age. Unpaired t-test was used to compare the potential difference between two groups and Pearson analysis was used to assess the correlation between age and PZP level.
