# Supplementary material for: A Novel Diagnostic Biomarker, PZP, for Detecting Colorectal Cancer in Type 2 Diabetes Mellitus Patients Identified by Serum-Based Mass Spectrometry
Source: Front Mol Biosci. 2021 Nov 30;8:736272. doi: 10.3389/fmolb.2021.736272 (PMC8670180; doi:10.3389/fmolb.2021.736272)
Supplement: Supplementary file 1 [file Presentation1.ZIP › Fig S4.docx]

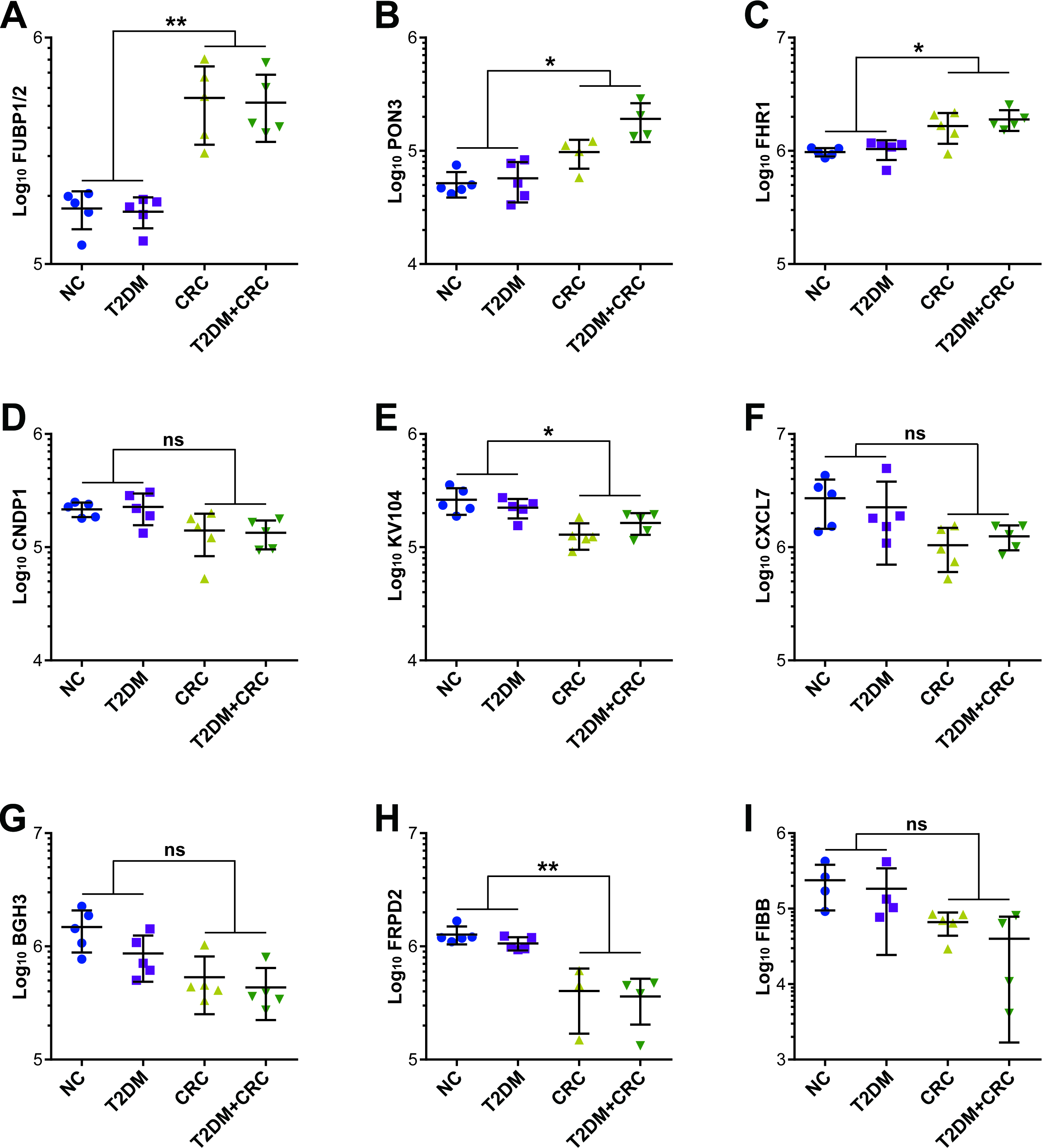


Figure S4. Statistical graphs of 9 proteins levels shared between the T2DM-CRC and CRC groups. Unpaired t-test was used to compare the potential difference.
