# Supplementary material for: A Novel Diagnostic Biomarker, PZP, for Detecting Colorectal Cancer in Type 2 Diabetes Mellitus Patients Identified by Serum-Based Mass Spectrometry
Source: Front Mol Biosci. 2021 Nov 30;8:736272. doi: 10.3389/fmolb.2021.736272 (PMC8670180; doi:10.3389/fmolb.2021.736272)
Supplement: Supplementary file 1 [file Presentation1.ZIP › Tab S1.docx]

Table S1. The baseline information of participants in the discovery cohort.

| Group | Gender | Age (years) | AFP (ng/ml) | CEA (ng/ml) | CA125 (U/ml) | CA199 (U/ml) | FBG (mmol/L) |
| --- | --- | --- | --- | --- | --- | --- | --- |
| NC |  |  |  |  |  |  |  |
| 1 | female | 70 | 1.6 | 1.9 | 10.2 | 1.7 | 5.5 |
| 2 | male | 64 | 1.9 | 1.3 | 9.6 | 2.8 | 4.6 |
| 3 | female | 35 | 2.3 | 1.0 | 8.3 | 3.2 | 4.7 |
| 4 | female | 64 | 4.1 | 2.4 | 7.6 | 0.8 | 5.2 |
| 5 | male | 43 | 3.7 | 3.1 | 6.3 | 0.8 | 4.5 |
| CRC |  |  |  |  |  |  |  |
| 1 | female | 64 | 1.4 | 4.7 | 9.4 | 44.1 | 5.1 |
| 2 | female | 56 | 1.6 | 1.7 | 19.9 | 7.1 | 5.6 |
| 3 | male | 70 | 1.9 | 3.0 | 6.0 | 7.0 | 5.4 |
| 4 | male | 69 | 2.9 | 0.7 | 7.0 | 2.6 | 5.9 |
| 5 | male | 80 | 3.4 | 82.5 | 23.3 | 207.3 | 4.1 |
| T2DM |  |  |  |  |  |  |  |
| 1 | male | 52 | 2.8 | 2.9 | 8.2 | 0.8 | 9.1 |
| 2 | male | 42 | 1.8 | 1.4 | 6.3 | 0.8 | 8.2 |
| 3 | male | 49 | 5.1 | 1.1 | 9.3 | 4.1 | 7.8 |
| 4 | female | 69 | 4.0 | 1.2 | 3.6 | 7.1 | 6.2 |
| 5 | male | 48 | 4.3 | 3.7 | 7.6 | 13.1 | 8.8 |
| T2DM+CRC | |  |  |  |  |  |  |
| 1 | female | 69 | 2.5 | 1.7 | 6.1 | 6.9 | 6.3 |
| 2 | female | 57 | 1.8 | 0.9 | 12.2 | 2.0 | 7.5 |
| 3 | female | 55 | 2.5 | 17.9 | 14.9 | 12.2 | 10.6 |
| 4 | male | 59 | 0.9 | 2.8 | 5.7 | 25.4 | 7.0 |
| 5 | male | 72 | 2.6 | 3.6 | 7.6 | 4.8 | 5.4 |
